# Few changes in native Australian alpine plant morphology, despite substantial local climate change

**DOI:** 10.1002/ece3.7392

**Published:** 2021-04-02

**Authors:** Meena S. Sritharan, Frank A. Hemmings, Angela T. Moles

**Affiliations:** ^1^ Fenner School of Environment & Society ANU College of Science Australian National University Acton ACT Australia; ^2^ Evolution & Ecology Research Centre School of Biological, Earth and Environmental Sciences UNSW Sydney Sydney NSW Australia

**Keywords:** alpine vegetation, climate change, leaf morphology, plant traits, rapid evolution, Southern Hemisphere

## Abstract

Rapid evolution is likely to be an important mechanism allowing native species to adapt to changed environmental conditions. Many Northern Hemisphere species have undergone substantial recent changes in phenology and morphology. However, we have little information about how native species in the Southern Hemisphere are responding to climate change. We used herbarium specimens from 21 native alpine plant species in Kosciuszko National Park, Australia, to make over 1,500 measurements of plant size, leaf thickness, leaf mass per area, leaf shape, and leaf size across the last 126 years. Only two out of 21 species (9%) showed significant changes in any of the measured traits. The number of changes we observed was not significantly different to what we would expect by chance alone, based on the number of analyses performed. This lack of change is not attributable to methodology—an earlier study using the same methods found significant changes in 70% of species introduced to southeast Australia. Australia's native alpine plants do not appear to be adapting to changed conditions, and because of the low elevation of Australia's mountains, they do not have much scope for uphill migration. Thus, our findings suggest that Australia's native alpine plants are at even greater risk in the face of future climate change than was previously understood.

## Graphical abstract

This study is the first test of whether alpine plants in the Southern Hemisphere have changed their morphology in response to climate change. Using herbarium specimens from 21 native alpine plant species in Kosciuszko National Park, Australia, we found that only 9% of the species showed any significant changes in plant or leaf traits over the last 126 years, standing in stark contrast to the number of changes recorded in introduced plants (up to 70%) using the same methods. A lack of morphological change combined with limited migration options suggests that native alpine plants may face a bleak future.

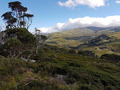

## 1 INTRODUCTION

Changes in plant growth and distribution have been observed as an effect of global anthropogenic climate change. Some authors have suggested that species will be unable to respond to the speed and magnitude of anthropogenic climate change, leaving them vulnerable to extinction (Hoffmann & Sgrò, 2011). However, recent studies have shown that rapid evolutionary change can occur over just a few generations (Franks & Weis, 2008; Hoffmann & Sgrò, 2011). Introduced plants frequently undergo rapid evolution when subjected to new environmental conditions (Brandenburger et al., 2019; Buswell et al., 2011; Thompson, 1998), but relatively few native plants have been observed to undergo rapid evolution, and the evidence we do have is predominantly from the Northern Hemisphere (Fitter & Fitter, 2002; Franks & Weis, 2008; Kudo et al., 1999; Parmesan, 2006). We currently have little knowledge of whether native plants, particularly those in the Southern Hemisphere, are responding to changes in climate.

Environmental conditions differ in several ways between the Southern Hemisphere and the Northern Hemisphere. Differences such as a smaller annual temperature range and higher minimum temperatures in the Southern Hemisphere (Chown et al., 2004; Jones et al., 1999; Harris et al., 2008), combined with a faster rate of climate change in the Northern Hemisphere (Easterling et al., 1997), may produce substantial differences between how Northern Hemisphere and Southern Hemisphere species respond to climate change (Chown et al., 2004). For instance, the Northern Hemisphere has seen greater recent changes in terrestrial net primary productivity and evapotranspiration than has the Southern Hemisphere (Li et al., 2016).

A few studies in the Southern Hemisphere have looked at the effect of climate change in native plants (Gallagher et al., 2009; Guerin et al., 2012) with some native species shown to be capable of rapid changes in morphology (Buswell et al., 2011; Guerin et al., 2012). For instance, Buswell et al., (2011) found one of the four studied native species had decreased in height over the last century, but no changes in leaf shape or leaf area were found in the other three native species. Guerin et al., (2012) found decreasing leaf width in native *Dodonaea viscosa* over 127 years, but whether this change is related to climate change is under debate (Duncan, 2013).

Like many ecosystems worldwide, alpine regions face unprecedented ecological change and potential biodiversity loss as a consequence of climate change (Nicotra et al., 2016). Higher‐than‐average temperature increases in alpine areas in the past century (Theurillat & Guisan, 2001) have led to altered distributions and changes in the morphology of plants in Northern Hemisphere alpine regions (Gottfried et al., 2012). For instance, plants with higher leaf mass per area and thicker leaves in the alpine belt of Caucasus Mountains, Russia, have increased in abundance in response to climate warming (Soudzilovskaia et al., 2013). However, there is no information on whether alpine plants in the Southern Hemisphere have changed in morphology over time in response to recent warming. Our study aims to address this knowledge gap.

The first trait we investigated was plant size. Plant size is a crucial component of plant ecological strategy, central in determining how a species lives, grows, and reproduces (Moles, 2018). As temperatures increase and growing seasons become longer due to earlier snowmelt (Jonas et al., 2008), alpine plants are expected to increase their growth rate. This prediction is supported by data from tundra ecosystems in the Northern Hemisphere, where increases in plant height of deciduous shrubs increased with climatic warming over time (Gamache & Payette, 2004; Walker et al., 2006). We therefore predicted that plant size would increase over time.

Second, we asked how leaf thickness and leaf mass per area (LMA) have changed over time. These traits are key elements of a plant's leaf economic strategy, affecting strategies for resource acquisition and use (Givnish, 1979; Wright et al., 2004). Thick leaves and/or leaves with high LMA tend to have long life spans associated with high construction costs and low maximum photosynthetic rates (Westoby et al., 2002; Wright et al., 2004). It has been shown that plants can respond to changes in climate with changes in LMA (Poorter & De Jong, 1999; Rosbakh et al., 2015), although there is an inconsistent relationship between temperature and LMA across studies (Moles, 2018). Climatic factors can also influence leaf thickness because thicker leaves are more heat‐tolerant and therefore commonly found in hot, open environments (Groom et al., 2004). We predicted that alpine plants would have increased LMA and increased leaf thickness in order to take advantage of the longer growing seasons associated with increasing temperatures.

Third, we asked whether leaf size and/or leaf shape changed over time. Leaf size traits are sensitive to climate and altitude, and correlate strongly with temperature from global to local scales (Moles, 2018). Small and narrow leaves improve cooling in warm conditions and have rapid rates of heat convection (Geller & Smith, 1982; Parkhurst & Loucks, 1972) allowing plants to shed heat through sensible heat loss (Yates et al., 2010). Larger leaves, which have thicker boundary layers, have greater difficulty losing heat under warmer conditions and absorbing heat from their surroundings. Consequently, larger leaves are more vulnerable to damage on colder nights (Wright et al., 2017), a concern for alpine plants experiencing decreases in snow cover (McGowan et al., 2018). As wider leaves experience higher than average temperatures, consequently facing greater day heat and cold night stresses, we predicted that alpine species would show a shift toward smaller, narrower leaves in response to increasing temperatures.

Finally, we asked whether native alpine species were showing fewer changes over time than introduced species globally. Previous research using herbarium specimens has found rapid morphological trait changes in introduced plants in both the Northern Hemisphere and the Southern Hemispheres (Buswell et al., 2011; Dalrymple et al., 2015; Flores‐Moreno et al., 2015; Guerin et al., 2012). In all these studies, native species showed fewer trait changes than introduced species. Therefore, we predicted that this would also be the trend for the native alpine plants in our study.

In summary, our hypotheses were that over time, Australian alpine plants would show.

1. increased plant height,
2. increased leaf thickness and leaf matter per area,
3. smaller, narrower leaves, and
4. fewer changes than introduced plants globally.

## 2 METHODS

### 2.1 Study site

**FIGURE 1:**
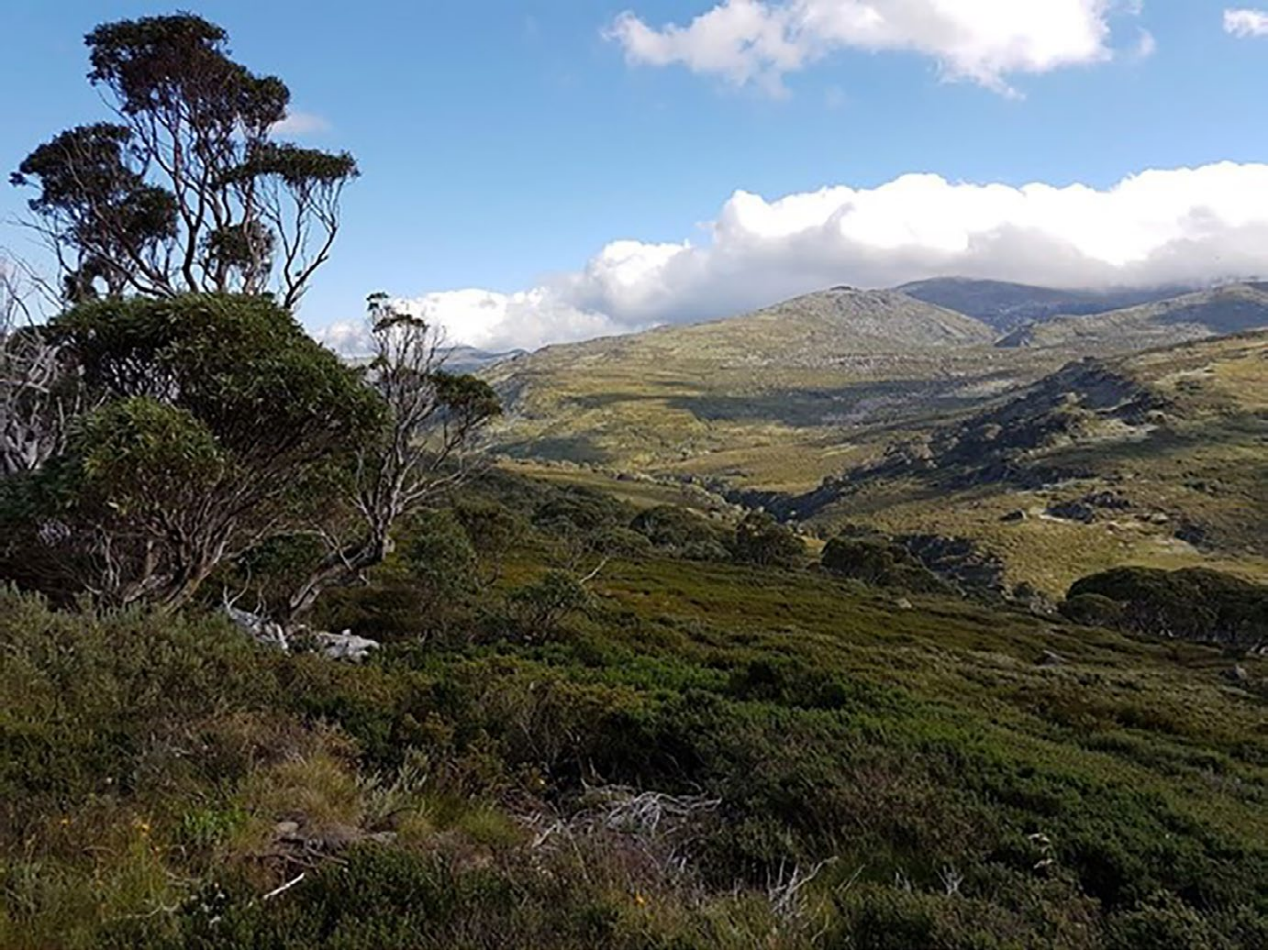
Kosciuszko National Park, Australia. Photo by Angela Moles (February 2017)

Our study was performed in Kosciuszko National Park, in southeastern Australia (Figure 1). Plants were sampled from the tree line at 1,800 m (Costin et al., 2000) to 2,228 m, the summit of Australia's highest peak, Mount Kosciuszko. The area is of high conservation value due to an abundance of endemic fauna and flora (Kirkpatrick, 2002; Pickering & Buckley, 2003). With alpine regions being one of the least resistant ecosystems to the effects of climate change (Theurillat & Guisan, 2001), the region has also been identified as highly vulnerable, with many species predicted to decline or face extinction in the future (Hennessy et al., 2008).

**FIGURE 2:**
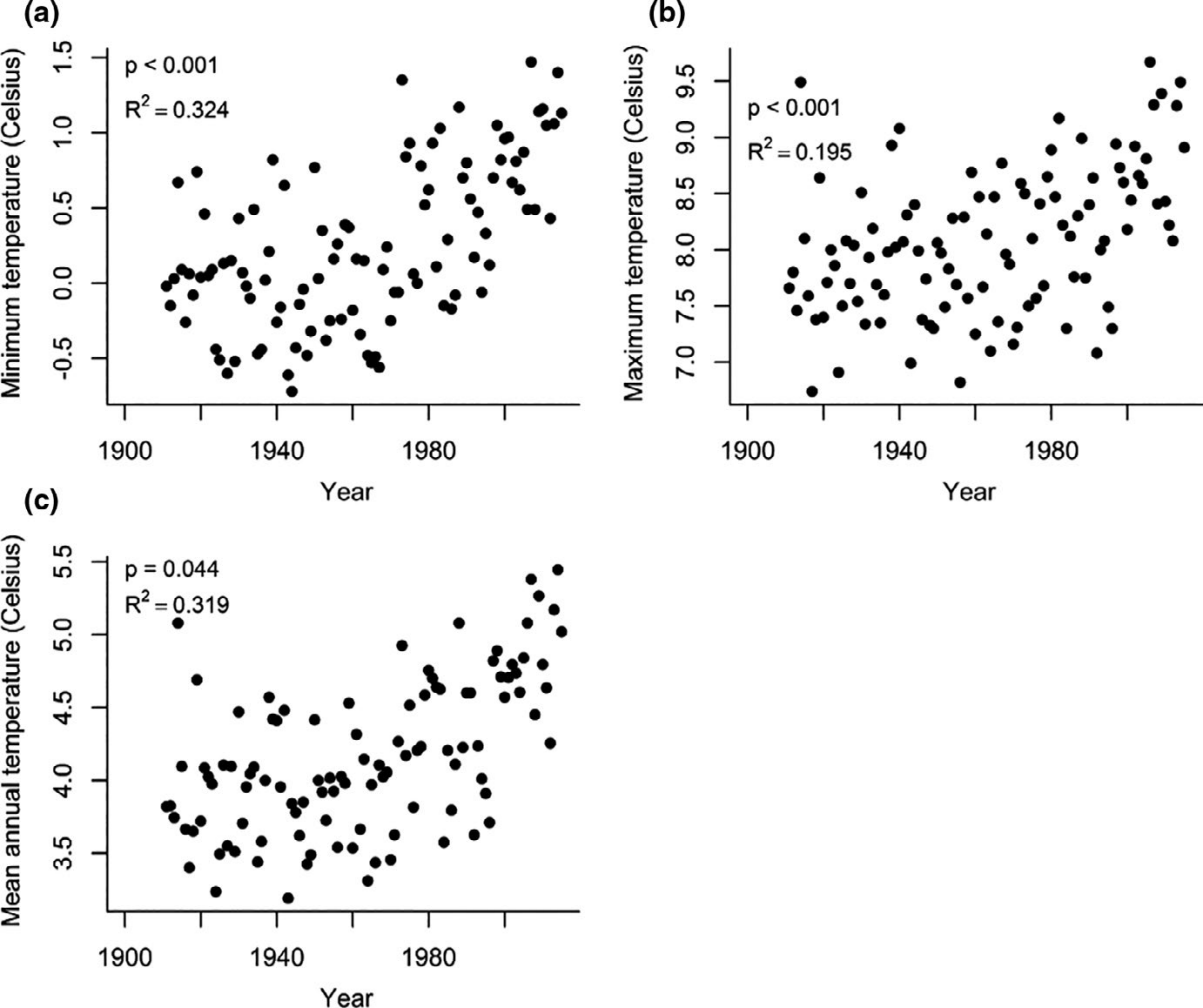
Annual maximum (a), minimum (b), and mean (c) temperatures (°C) above 1800m in Kosciuszko National Park between 1911 and 2015. Data were obtained from the Australian Water Availability Project (http://www.csiro.au/awap/) dataset (details available in Appendix S1

The area above the tree line in Kosciuszko National Park has faced a rapid increase in temperature over time (Figure 2; details in Appendix S1) with an associated extension of the growing season (Jonas et al., 2008) and earlier snowmelt (Hennessy et al., 2008).

### 2.2 Species

We obtained a list of all native alpine plant species present above 1,800 m and within a 5 km radius from the summit of Mount Kosciuszko from the Atlas of Living Australia (http://www.ala.org.au/). For inclusion in our study, the following three criteria needed to be satisfied: Species had to have at least 15 independent herbarium specimens (i.e., specimen collections made in different years); specimens had to have three intact, mature, nonsenescing leaves; and information on the year of collection and altitude (or sufficient location data to estimate altitude to within 50 m) needed to be available. These selection criteria yielded 21 species from 14 families.

### 2.3 Specimen sampling

We used a combination of historic and newly collected (modern) herbarium specimens. Historic specimens, collected between 1890 and 2016, were sourced from the John T. Waterhouse Herbarium at UNSW Sydney (UNSW), Downing Herbarium at Macquarie University (MQU), National Herbarium of New South Wales at the Royal Botanic Gardens Sydney (NSW), and the Australian National Herbarium at CSIRO in Canberra (CANB). Five modern specimens of each species were collected at a range of altitudes above the tree line (1,800 m) in February 2017. Including these modern specimens helped ensure that our dataset reached the present day and gave us power to account for the role of altitude in shaping plant traits. We were careful to minimize differences between modern and historic collections to avoid the introduction of sampling biases. All modern and historic herbarium specimens used were collected within a five kilometer radius from the summit of Mount Kosciuszko and from sites above 1,800 m. Historic specimens tend to be collected in locations easily accessed from roads and walking trails (Daru et al., 2018), so we focused our modern collection along the major roads and walking tracks in the sample area. To reduce possible differences in collector biases between historic and modern specimens, all modern collections were made by F. Hemmings, curator, and main collector for the John T. Waterhouse Herbarium, and this collector was not told the purpose of the collections or the aim of our study until after the specimen collecting had been completed. Modern specimens were lodged at the John T. Waterhouse Herbarium (UNSW), with duplicates lodged at NSW and CANB.

### 2.4 Plant trait measurement

Due to the varying conditions of herbarium specimens, it was not possible to measure every trait on every species. However, we measured all the possible traits available for each species. We counted multiple specimens on the same herbarium sheet as separate observations only if we were sure they were separate individuals (otherwise they were treated as one individual). We used 150 mm digital calipers to measure leaf length, leaf width, and plant height. For species that had large and curled leaves, we used a 40 cm ruler marked to 0.5 mm and polypropylene garden twine. Specimens/leaves were not moved from the herbarium sheets for measurements of plant size or leaf size and shape, but we had to remove leaves from specimens to make measurements of leaf mass per unit area and leaf thickness. The details on how leaf length, width, and height were measured on each plant species are provided in Table S1, Appendix S2.

To quantify plant size, we measured plant height or rosette width. Height was measured from the base of the stem where the root began to the top of the main photosynthetic tissue, excluding inflorescences and any stems extending above the main foliage (following Buswell et al., 2011). We were only able to measure plant height for *Oreomyrrhis pulvinifica*, as all other species either (a) had rosette growth forms,(b) were larger than a herbarium sheet (for which collectors might have biased collections toward smaller specimens that fit on herbarium sheets), or (c) had specimens that did not include roots. Rosette width was determined as the maximum diameter of the rosette.

Next, we measured leaf thickness and leaf mass per area (LMA) according to standard protocols (Pérez‐Harguindeguy et al., 2013). Because these traits require destructive sampling of historical specimens, we were limited to measuring only one leaf per specimen and to sampling species with numerous small leaves. Leaf thickness was derived by measuring three points on each lamina using a dial gauge (Type 50, Mercer) and then averaging the results. Leaf area (including the petiole) was measured using ImageJ (Rasband, 2013). Leaves were then dried at 50°C for 24 hr. After cooling in a desiccator, leaves were weighed to the nearest hundredth of a gram using an analytical balance (Mettler Toledo XS). LMA was calculated as the dry weight of the leaf divided by its area. Leaf area may decrease slightly through the drying process (Queenborough & Porras, 2014), so to make modern data as comparable to the historic data as possible and reduce biases, all leaf area measurements on both modern and historic specimens were made on dried, pressed leaves.

Finally, we measured leaf size and leaf shape following Buswell et al., (2011). For the six species collected for LMA analysis, leaf size was assessed as a measure of leaf area. For species that could not be measured for leaf area (e.g., for species with leaves that had curled margins or were frequently found to be damaged), we used leaf length as a proxy for leaf size. Leaf shape was calculated as the ratio of leaf width to leaf length. Leaf width was measured as the maximum diameter of the largest imaginary circle that could be fitted on the leaf. Leaf length was measured as the longest distance between the tip and the base of the leaf, excluding the petiole. We measured leaf length and width on 3–10 nonsenescing, mature leaves, depending on the number of available intact leaves for each specimen (Table S1, Appendix S2). Measurements were taken using either digital calipers to 0.01 mm accuracy (150 mm; Vernier) or a ruler. For leaves that had curled during preservation, we used a piece of string and a ruler to estimate the original size.

### 2.5 Statistical analyses

To analyze change in traits over time, we fitted linear mixed‐effects models for each species. Nested random factors were used to account for multiple individuals collected at one site and placed on a single herbarium sheet (because specimens collected at the same site are more likely to be similar than individuals selected randomly from the population).

Models also included terms for altitude. We first fitted a model including an interaction term between “year” and “altitude,” and if there was no significant interaction (*p* > 0.05), we removed the interaction effect and reran the analysis. We also considered the possibility that specimens might show temporal autocorrelation. Each model was run with and without a coefficient to account for temporal autocorrelation. In cases where there was no significant difference between these models (ANOVA, alpha = 0.05), we present results from models without temporal autocorrelation. In cases where there was a significant difference, we present results from the analysis with the lowest Akaike's information criterion (AIC) value.

All analyses were conducted in R 3.3.1 (R Development Core Team, 2015). Models were fitted using the R package *lme4* (Bates et al., 2015). Pseudo‐*R* ^2^ values were obtained using the *Mumin* package (Barton, 2019).

### 2.6 Data considerations

All trait data were log_10_‐transformed before analysis. Nevertheless, the results of analyses on untransformed data are qualitatively similar to those of analyses on log_10_‐transformed data (Table S2, Appendix S3).

In four analyses, our datasets included points isolated temporally from the next closest data point by more than 30 years. In these cases, analyses were run with and without the temporal outliers. In no case did exclusion of these temporal outliers qualitatively change the outcome of the analysis. Thus, it does not seem that inclusion of temporally isolated data points artificially strengthened or weakened any of our results.

To determine whether our choice of analysis might have affected the number of significant changes detected, we also conducted weighted models (following Buswell et al., 2011). Results using the weighted models method were analogous to those from our nested approach, except for one trait in one species (rosette width in *Plantago muelleri*; Table S2, Appendix S3). Thus, the differences in analysis methods are not likely to be the cause of any differences in results between the two studies.

As several independent statistical tests were performed, there was a high chance of false‐positive results and so we used sequential Bonferroni corrections for each significant change in plant traits (Abdi, 2010).

## 3 RESULTS

**FIGURE 3:**
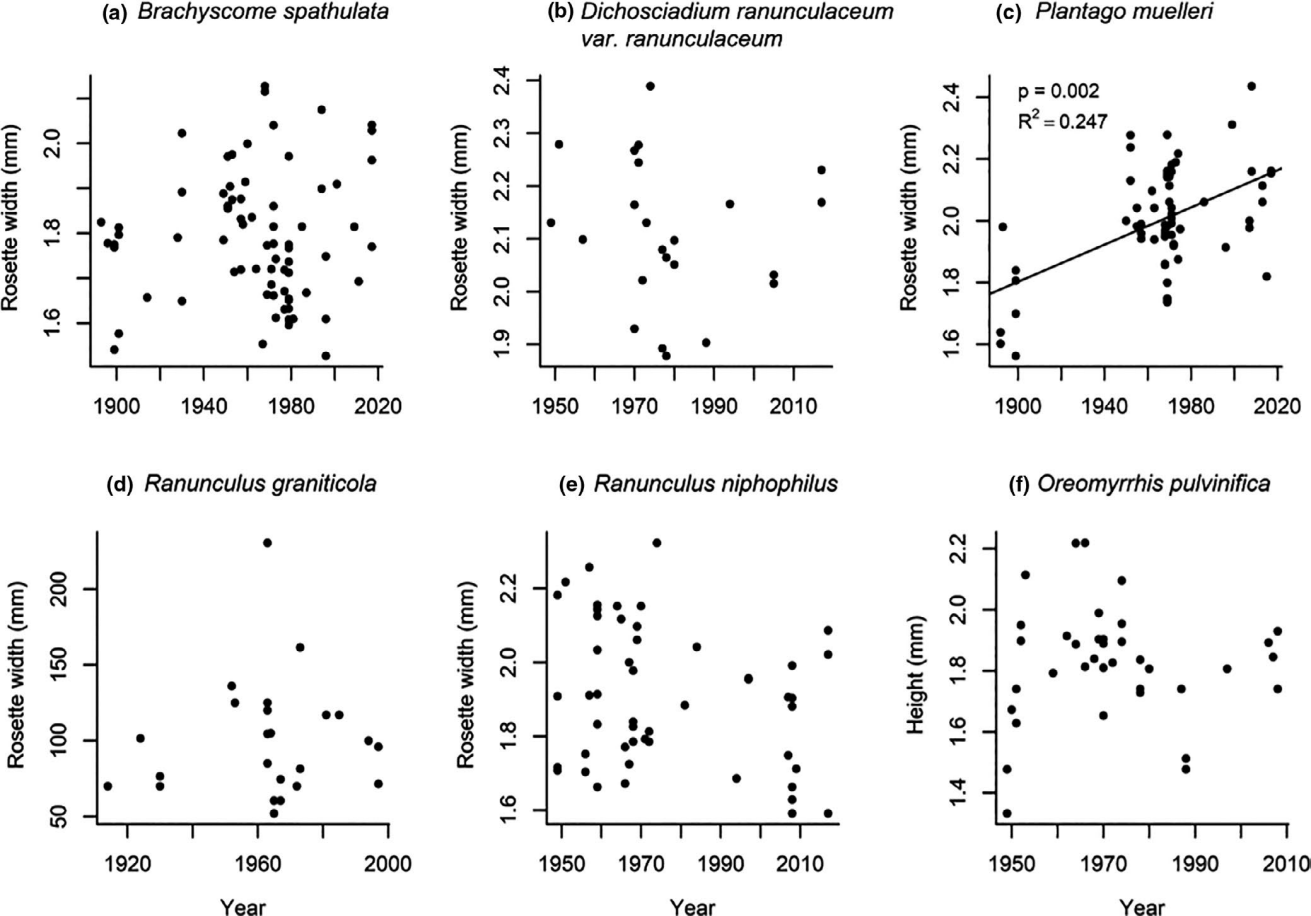
Plant size (rosette width, a–e; plant height, f) measured on six native alpine plant species in Kosciuszko National Park. Each data point represents one individual. Values for *p* and *R* ^2^ are given where we found significant change over time. Only *Plantago muelleri* showed significant changes, with an increase in rosette width over time

Only one of the six species for which plant size could be quantified showed a significant change over time (Figure 3). The increase in rosette width in *P. muelleri* was in a direction consistent with our prediction and was substantial in magnitude (21% increase over 125 years; *p* = 0.002, *R* ^2^ = 0.247).

**FIGURE 4:**
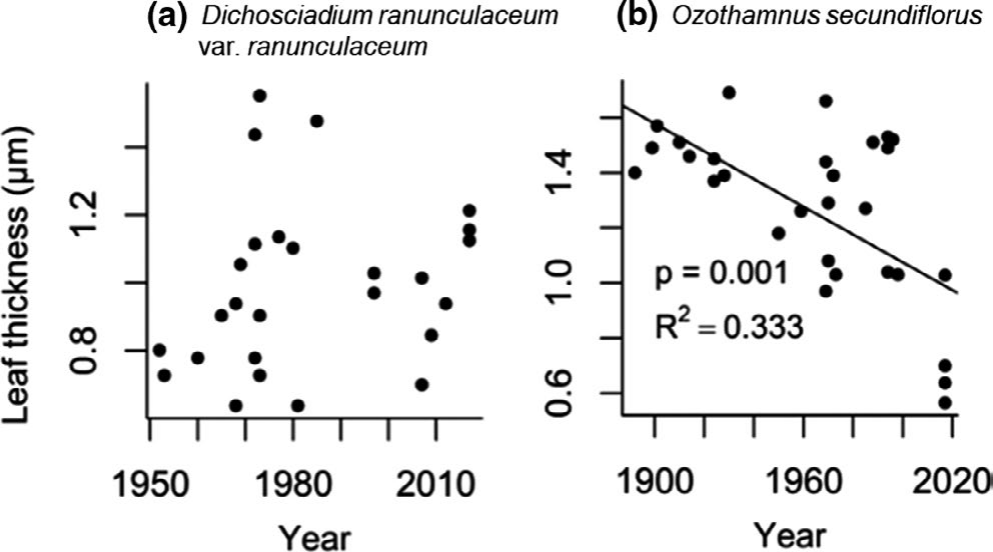
Leaf thickness measured on two native alpine plant species in Kosciuszko National Park. Each data point represents one individual. Values for p and *R* ^2^ are given where we found significant change over time. Only *Ozothamnus secundiflorus* showed significant changes, with a decrease in leaf thickness over time

**FIGURE 5:**
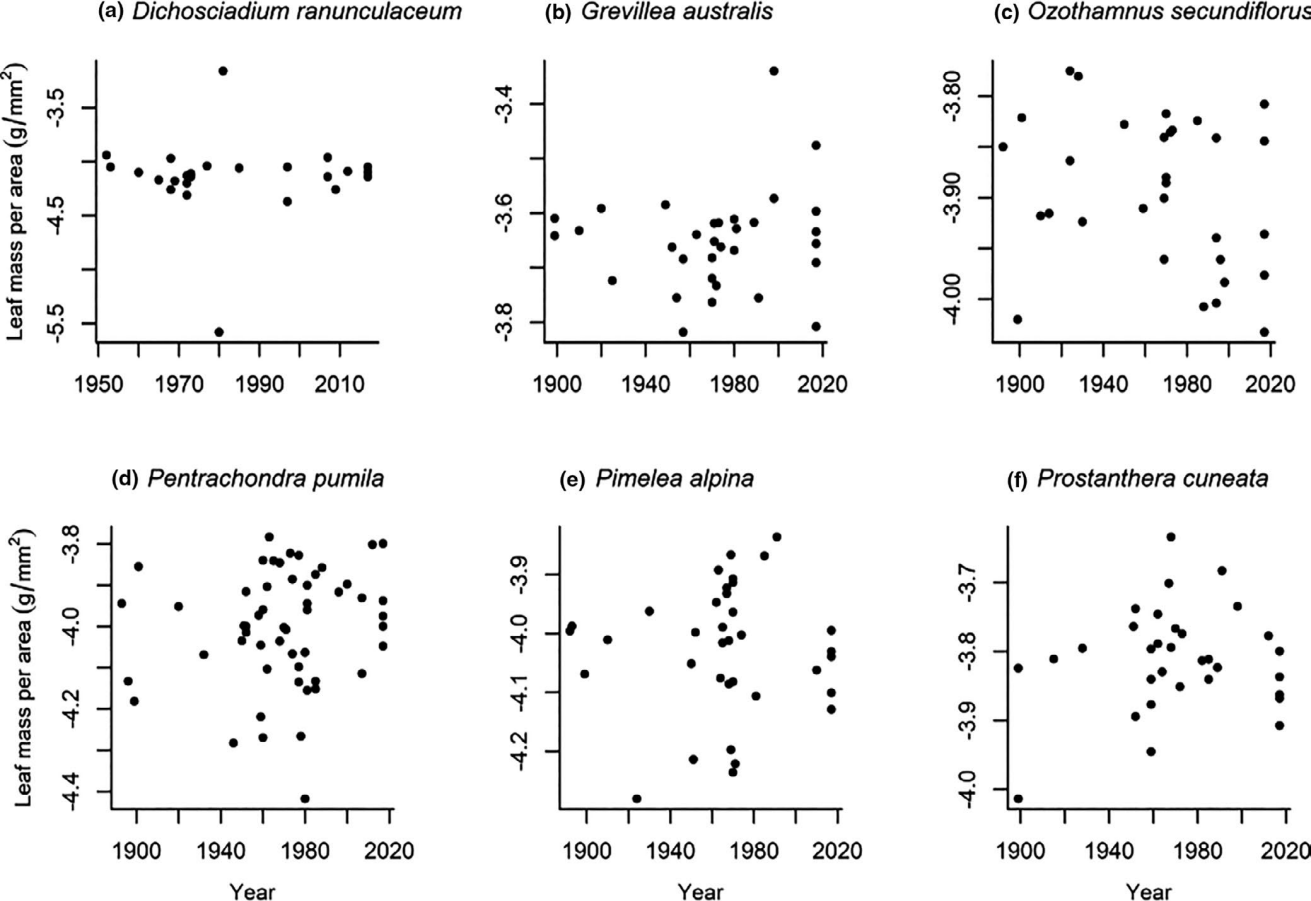
Leaf mass per area (LMA) measured on six native alpine plant species in Kosciuszko National Park. Each data point represents one individual. No significant changes in LMA were recorded

One of the two species examined for leaf thickness showed a significant change over time. Leaf thickness in *Ozothamnus secundiflorus* decreased by 38% over a 125‐year period (*p* = 0.010, *R* ^2^ = 0.149, Figure 4). However, the direction of this change was not consistent with our prediction. No species showed a significant change in LMA (Figure 5).

**FIGURE 6:**
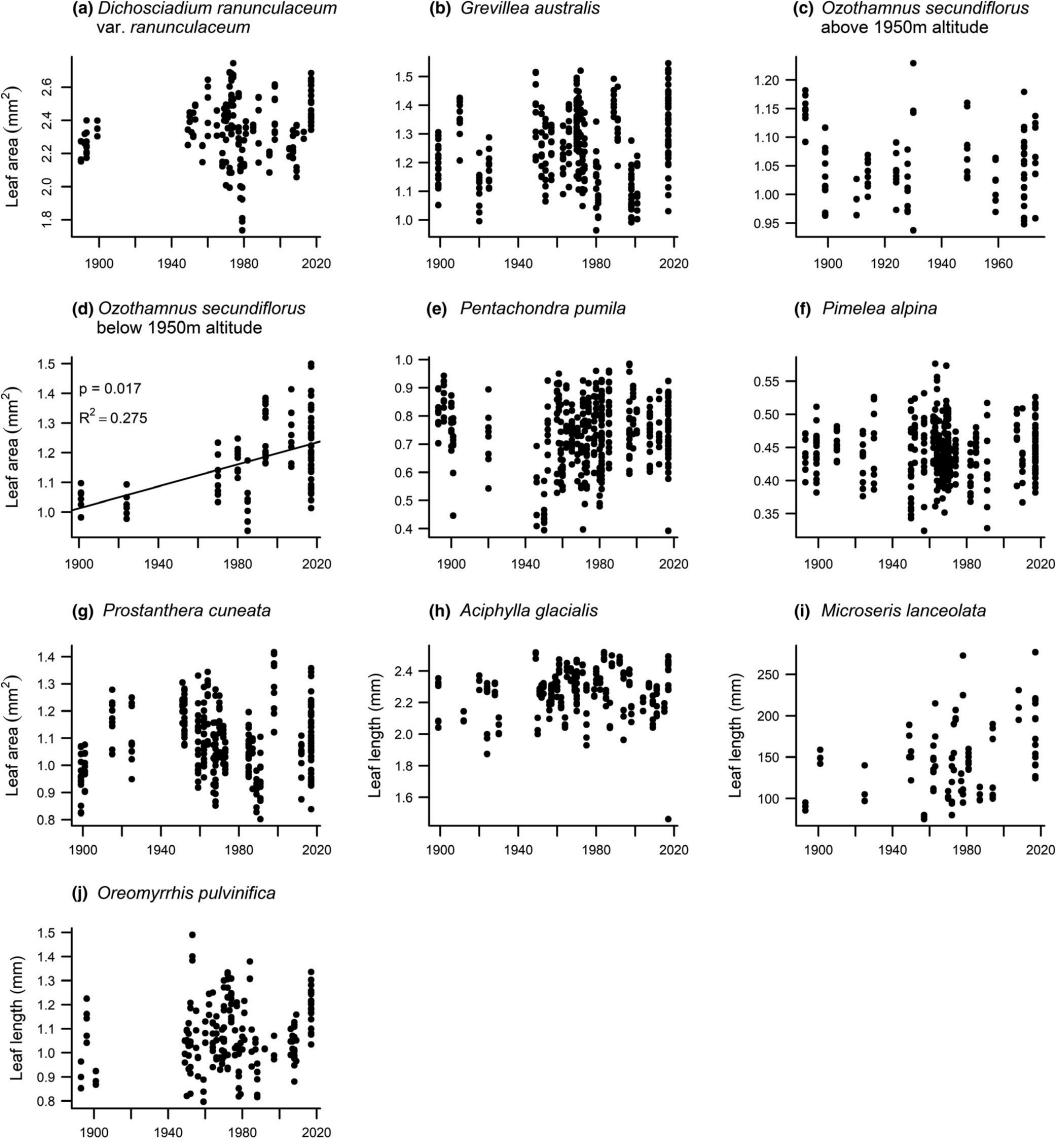
Leaf size (area, a–g; length, h–j) measured on ten native alpine plant species in Kosciuszko National Park. Each data point represents one individual. Values for p and *R* ^2^ are given where we found significant change over time. For *Ozothamnus secundiflorus*, there was a significant interaction effect between year and altitude in the analysis of leaf area (*p* < 0.01)

**FIGURE 7:**
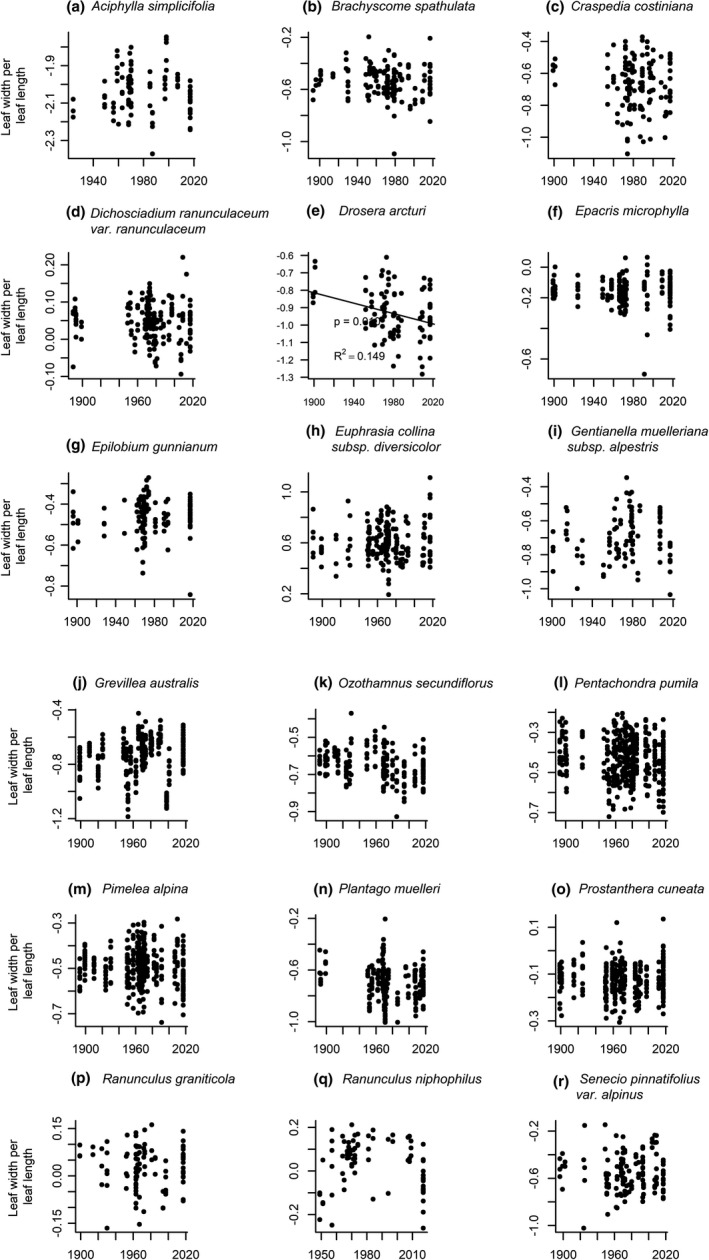
Leaf shape (leaf width per leaf length) measured on 18 native alpine plant species in Kosciuszko National Park. Each data point represents one individual. Values for *p* and *R* ^2^ are given where we found significant change over time. Only *Drosera arcturi* showed significant changes, with a decrease in leaf width per leaf length over time

No significant changes were observed in leaf size over time (Figure 6). However, for one species, *O. secundiflorus*, there was a significant interaction effect between year and altitude in the analysis of leaf area (*p* < 0.01) indicating that there was a change in the altitude at which this species had been collected over time (Figure S1, Appendix S4). To avoid confounding due to changing altitude, we split the data into two altitudinal bands. There was no significant change over time in the leaf area of *O. secundiflorus* specimens collected above 1,950 m (Figure 6c). There was a trend for specimens collected below 1,950 m to increase in leaf area over time (*p* = 0.002, *R* ^2^ = 0.245, Figure 6d), but this relationship did not remain significant after sequential Bonferroni correction. No significant changes were observed in leaf shape over time (Figure 7).

Overall, only three out of the 21 species we sampled showed changes in any measured trait over time (Table S2, Appendix S3). After sequential Bonferroni corrections, only two of these results remained significant (9%). A binomial test (following Buswell et al., 2011) showed that the probability of observing two significant results over 45 analyses due to chance alone was high (*p* > 0.05).

The proportion of species showing significant trait changes over time was substantially lower in the present study than in several previous studies that applied the same methods. The proportion of native Australian alpine species that displayed significant changes in traits over time was significantly lower (*χ* ^2^ = 16.37, *df* = 1, *p* < 0.001) than the 70% of sexually reproducing introduced species that showed change over time in Australia (Buswell et al., 2011). It was also significantly lower (*χ* ^2^ = 4.341,

*df* = 1, *p* = 0.037) than the 50% of clonally reproducing introduced species that showed change over time in New Zealand and Australia (Dalrymple et al., 2015) and the 66% (though this was based on only three species) of sexually reproducing introduced species that showed significant change over time in the United Kingdom (*χ* ^2^ = 6.17, *df* = 1, *p* = 0.013) (Flores‐Moreno et al., 2015). However, the proportion of changes in native alpine species in the present study was not significantly different to the proportion of changes in the Australian native species included in Buswell et al.,'s (2011) study as a control group (one out of five native species showed significant change over time; *χ* ^2^ = 0.24, *df* = 1, *p* = 0.62).

## 4 DISCUSSION

Only two out of the 21 native Australian alpine plant species we sampled showed changes through time in the morphological traits we measured in this study. Of course, we have sampled only a limited subset of species and considered only a few traits that we chose because we thought they were likely to have been under selective pressure to change in response to changes in climate. Notwithstanding these limitations, our data suggest that Australian native species are undergoing fewer changes than are introduced species. Our results are in line with previous studies finding few native Australian plants showing rapid changes in morphology (Buswell et al., 2011; Guerin et al., 2012) in comparison with introduced species (Buswell et al., 2011; Dalrymple et al., 2015). We can think of three possible reasons for a difference in the rate of change between native and introduced species.

First, species that have been introduced to a new range are often exposed to very different abiotic conditions than those in their native range and experience a new suite of biotic interactions. This may lead to more intense selective pressures than are faced by native species exposed to climate change. One way to test this idea would be to ask whether the amount of change species undergo when introduced to a new range is correlated with the amount of difference in climate between the home range and the introduced range.

Second, introduced species might be more likely to possess life‐history traits and strategies that facilitate rapid evolution than are native species (Davidson et al., 2011). For example, introduced flora that have evolved rapidly to changing environmental conditions are often annual species, have a short juvenile period, or display rapid population growth (Dalrymple et al., 2015; Davidson et al., 2011). There are very few annual species in the Australian alpine zone, and all 21 of our study species are perennial. This idea could be tested by compiling a large dataset of species change through time and asking whether plant traits explain a significant proportion of the variation in the amount of change species have undergone.

Third, the low rate of change in native Australian species could reflect differences between Australia and other parts of the world. Australia is renowned for having a variable climate and having unique climatic differences to other continents (Cleverly et al., 2019; Murphy & Timbal, 2008). Long‐lived species that evolved under high levels of climatic variability may, in the short to medium term, be pre‐adapted to the climatic changes expected (Adler et al., 2006; Morris et al., 2008; Venn et al., 2009). Species with adaptation to high climatic variability may experience an evolutionary lag in responding to rapid changes in climate (Wilczek et al., 2014). Important next steps include determining whether the amount of change in native species from different regions worldwide is less in regions with high climate variability.

One of the two changes we did see (an increase in rosette width in *P. muelleri*) was in line with our predictions, but the other (an increase in leaf size in *O. secundiflorus*) was counter to what we predicted. That is, our ability to use our knowledge of relationships between plant traits and temperature to predict how species might respond to climate change is extremely weak. Further, even with the benefit of hindsight, it is difficult to guess why these two species changed, while so many others did not. Gathering further empirical data on the ways native plant species are changing their morphology in response to climate change and testing hypotheses about what type of species are most likely to change are important goals for the future.

While our study did not specifically investigate elevational shifts, our data revealed that one species had changed in elevation over time. Contrary to predictions based on warming, *O. secundiflorus* had undergone a substantial downslope range shift (Figure S1, Appendix S4). This is not an isolated incident as several species have similarly experienced downslope range shifts globally (Lenoir et al., 2010), a movement inconsistent with the forecasted effects of species migrations moving to higher elevations with climate warming. A global review of literature by Parmesan and Yohe (2003) showed 20% of species experiencing range shifts have adjusted their ranges toward lower elevations or southern latitudes. Such observations challenge current assumptions of species migrating to remain areas with cooler temperatures, as is assumed in ecological niche models, which aim to predict future species distributions by combining present‐day distributions with environmental variables (Dullinger et al., 2012). The downslope migration of alpine plants may be partly explained by earlier snowmelt exposing alpine plants to colder temperatures (Briceño et al., 2014) or by cold air drainage (Dobrowski, 2011). Quantifying the frequency and direction of distribution changes in other native species in the Southern Hemisphere is an important direction for future research.

The low rate of change over time in alpine species observed in our study is of concern, as it suggests that in situ adaptation is not occurring despite substantial local climate change (Figure 2). The Australian alpine environment spans only 0.09% of Australia (Gallagher et al., 2009) and reaches a maximum elevation of just 2,228 m. Thus, even if alpine plant species were able to migrate fast enough to keep pace with climate change, they have limited space to colonize. A combination of limited migration options and a lack of morphological change suggests that native alpine plants may face a bleak future.

## CONFLICT OF INTEREST

The authors have no conflict of interest to declare.

## AUTHOR CONTRIBUTIONS

**Meena Sivagowre Sritharan:** Conceptualization (supporting); Data curation (lead); Formal analysis (lead); Investigation (lead); Methodology (equal); Project administration (equal); Resources (supporting); Validation (equal); Visualization (lead); Writing‐original draft (lead); Writing‐review & editing (equal). **Frank A. Hemmings:** Investigation (equal); Methodology (supporting); Project administration (supporting); Resources (equal); Writing‐review & editing (equal). **Angela T. Moles:** Conceptualization (lead); Data curation (supporting); Formal analysis (supporting); Funding acquisition (lead); Investigation (supporting); Methodology (equal); Project administration (equal); Resources (lead); Supervision (lead); Validation (equal); Visualization (supporting); Writing‐original draft (supporting); Writing‐review & editing (equal).

## ETHICAL APPROVAL

No ethics permit was required for this project. Fieldwork was conducted under Scientific Licence SL101764 from the New South Wales National Parks and Wildlife Service.

## Supporting information

Supplementary Material

## Data Availability

The full dataset is available at https://doi.org/10.5061/dryad.m0cfxpp34.
