# Supplementary material for: Few changes in native Australian alpine plant morphology, despite substantial local climate change
Source: Ecol Evol. 2021 Apr 2;11(9):4854–65. doi: 10.1002/ece3.7392 (PMC8093687; doi:10.1002/ece3.7392)
Supplement: Supplementary file 1 — Supplementary Material [file ECE3-11-4854-s001.docx]

SUPPLEMENTARY INFORMATION

# Few changes in native Australian alpine plant morphology, despite substantial local climate change

Meena Sivagowre Sritharan*^1, 2^ and Angela T. Moles^2^

**Appendix S1.** Collection of temperature data

**Appendix S2.** Measurement methods for leaf length, leaf width and plant size

**Appendix S3.** Linear models for all plant traits

**Appendix S4.** Altitudinal shift over time for *Ozothamnus secundiflorus*

## Appendix S1. Collection of temperature data

Data on minimum, maximum and mean annual temperature were extracted from the Australian Water Availability Project dataset (AWAP; <http://www.csiro.au/awap/>) by
A Pepler and LV Alexander (Bureau of Meteorology <http://www.bom.gov.au/>; pers. comm.) using the nearest five-kilometre grid box site to Mount Kosciuszko. Temperatures from the 1911-2015 were obtained in order to observe changes over time in Kosciuszko National Park, in areas above the tree line at 1800m.

The AWAP temperature data is known to contain inhomogeneities in the data. Therefore, to confirm the accuracy of the AWAP data we collected, we used a higher quality data set from nearby and analysed correlations in temperature anomalies. We first obtained trends and variability data from the Australian Climate Observations Reference Network – Surface Air Temperature (ACORN-SAT) dataset for the Cabramurra station, 60km away from our study area. We then analysed correlations in temperature anomalies for the AWAP and ACORN-SAT data because anomalies are known to be spatially coherent over long distances and are less affected by factors such as elevation (Trewin, 2013). Using the *RHtestsV4* package (Wang, 2008, Wang and Feng, 2013, Wang et al., 2007) in R (R Development Core Team, 2015), we found a high (p < 0.0001) correlation between the AWAP and ACORN-SAT data. This provided significant confidence in the accuracy of our AWAP data.

#### References

**R Development Core Team.** **2015**. R: A language and environment for statistical computing. *R Foundation for Statistical Computing, Vienna, Austria.*

**Trewin B.** **2013**. A daily homogenized temperature data set for Australia. *International Journal of Climatology,* **33**: 1510-1529.

**Wang X.** **2008**. Accounting for autocorrelation in detecting mean-shifts in climate data series using the penalized maximal *t* or *F* test. . *Journal of Applied Meteorology and Climatology,* **47**: 423-2444.

**Wang X, Feng Y.** **2013.** RHtestsV4 User Manual. Climate Research Division, Atmospheric Science and Technology Directorate, Science and Technology Branch, Environment Canada. <http://etccdi.pacificclimate.org/software.shtml>.

**Wang XL, Wen QH, Wu Y.** **2007**. Penalized maximal *t* test for detecting undocumented mean change in climate data series. *Journal of Applied Meteorology and Climatology,* **46**: 916-931.

## Appendix S2. Measurement methods for leaf length, leaf width and plant size

**Table S1.** Number and location of leaves measured, and measurement method used for leaf length, leaf width and plant size (where possible) for 21 native alpine plant species in Kosciuszko National Park.

| **Species** | **Number and location of leaves measured** | **Measurement method** | | |
| --- | --- | --- | --- | --- |
|  |  | **Leaf length** | **Leaf width** | **Plant size** |
| Apiaceae | | | | |
| *Aciphylla glacialis* | Five leaves, on second tallest sheathing base | 40cm ruler marked to 0.5mm and polypropylene garden twine | N/A | N/A |
| *Aciphylla simplicifolia* | Five leaves | 40cm ruler marked to 0.5mm and polypropylene garden twine | Stainless steel 150mm digital callipers |  |
| *Dichosciadium ranunculaceum* var. *ranunculaceum* | Three leaves, from left to right of rosette | Stainless steel 150mm digital callipers | Stainless steel 150mm digital callipers | Rosette width with 40cm ruler marked to 0.5mm |
| *Oreomyrrhis pulvinifica* | Three leaves – the second, third and fourth tallest leaves of specimen | Stainless steel 150mm digital callipers | N/A | Plant height - 40cm ruler marked to 0.5mm and polypropylene garden twine |
| Asteraceae | | | | |
| *Brachyscome spathulata* | Four leaves | Stainless steel 150mm digital callipers | Stainless steel 150mm digital callipers | Rosette width with digital callipers |
| *Craspedia costiniana* | Three to five leaves | 40cm ruler marked to 0.5mm and polypropylene garden twine | Stainless steel 150mm digital callipers | N/A |
| *Microseris lanceolata* | Three leaves | 40cm ruler marked to 0.5mm and polypropylene garden twine | N/A | N/A |
| *Ozothamnus secundiflorus* | Eight leaves, chosen from the lowest branch of specimen | Stainless steel 150mm digital callipers | Stainless steel 150mm digital callipers | N/A |
| *Senecio pinnatifolius* var*. alpinus* | Three leaves, beginning from the third leaflet down from inflorescence | Stainless steel 150mm digital callipers | Stainless steel 150mm digital callipers | N/A |
| Droseraceae | | | | |
| *Drosera arcturi* | Three mature leaves (excluding hairs) | Stainless steel 150mm digital callipers | Stainless steel 150mm digital callipers | N/A |
| Epacridaceae | | | | |
| *Epacris microphylla* | Ten leaves, from lowest branch present on specimen | Stainless steel 150mm digital callipers | Stainless steel 150mm digital callipers | N/A |
| *Pentachondra pumila* | Ten leaves, from lowest branch present on specimen | Stainless steel 150mm digital callipers | Stainless steel 150mm digital callipers | N/A |
| Gentianaceae | | | | |
| *Gentianella muelleriana* subsp*. alpestris* | Five leaves from the top of the specimen down | Stainless steel 150mm digital callipers | Stainless steel 150mm digital callipers | N/A |
| Lamiaceae | | | | |
| *Prostanthera cuneata* | Ten leaves, from the second last branch | Stainless steel 150mm digital callipers | Stainless steel 150mm digital callipers | N/A |

| Onagraceae | | | | |
| --- | --- | --- | --- | --- |
| *Epilobium gunnianum* | Five leaves | Stainless steel 150mm digital callipers | Stainless steel 150mm digital callipers | N/A |
| Plantaginaceae | | | | |
| *Plantago muelleri* | Five leaves | 40cm ruler marked to 0.5mm and polypropylene garden twine | Stainless Steel 150mm digital callipers | Rosette width with digital callipers |
| Proteaceae | | | | |
| *Grevillea australis* | Ten leaves, from the second last branch | Stainless steel 150mm digital callipers | Stainless steel 150mm digital callipers | N/A |
| Ranunculaceae | | | | |
| *Ranunculus graniticola* | Five leaves | Stainless steel 150mm digital callipers | Stainless steel 150mm digital callipers | 40cm ruler marked to 0.5mm and polypropylene garden twine |
| *Ranunculus niphophilus* | Three leaves | Stainless steel 150mm digital callipers | Stainless steel 150mm digital callipers | 40cm ruler marked to 0.5mm and polypropylene garden twine |
| Scrophulariaceae | | | | |
| *Euphrasia collina* subsp*. diversicolor* | Five leaves, measured from the middle of each stem present | Stainless steel 150mm digital callipers | Stainless steel 150mm digital callipers | N/A |
| Thymelaeaceae | | | | |
| *Pimelea alpina* | Ten leaves, from the second last branch | Stainless steel 150mm digital callipers | Stainless steel 150mm digital callipers | N/A |

## Appendix S3. Linear models for all plant traits

**Table S2.** Results of a nested random factors model (with both log_10_ transformed data and untransformed data) and a weighted analysis method (Buswell et al., 2011) to test the null hypothesis that changes in plant size, leaf thickness, LMA, leaf shape and leaf area have not changed over time. Species marked with an * indicate an interaction effect was observed between year and altitude, resulting in models being rerun with the data split at the altitude which influenced the interaction effect. Values in **bold** indicate a significant result (*p* < 0.05); values in ***bold italic*** indicate a significant result (p < 0.05) after a sequential Bonferroni correction; n = number of specimens.

| **Species** | **Linear mixed-effects model with nested factors** | | | | | | **Weighted analysis method** | | | **n** |
| --- | --- | --- | --- | --- | --- | --- | --- | --- | --- | --- |
|  | **Transformed data (log_10_)** | | | **Untransformed data** | | |  |  |  |  |
|  | **p** | **R^2^** | **Slope** | **p** | **R^2^** | **Slope** | **p** | **R^2^** | **Slope** |  |
| Plant size | | | | | | | | | | |
| *Brachyscome spathulata* | 0.310 | 0.030 | 0.001 | 0.271 | 0.047 | 0.118 | 0.613 | 0.027 | <0.001 | 46 |
| *Dichosciadium ranunculaceum* var. *ranunculaceum* | 0.950 | 0.124 | 0.125 | 0.864 | 0.063 | 0.006 | 0.908 | 0.175 | <0.001 | 25 |
| *Oreomyrrhis pulvinifica* | 0.986 | <0.001 | <0.001 | 0.669 | 0.010 | -0.148 | 0.620 | 0.013 | -0.150 | 29 |
| *Ranunculus niphophilus* | 0.152 | 0.088 | -0.002 | 0.137 | 0.099 | -0.498 | 0.058 | 0.095 | -0.002 | 31 |
| *Ranunculus graniticola* | 0.521 | 0.077 | 0.001 | 0.589 | 0.055 | 0.203 | 0.471 | 0.087 | 0.001 | 26 |
| *Plantago muelleri ** | ***0.002*** | 0.247 | 0.003 | ***0.007*** | 0.183 | 0.583 | Interaction effect (altitude:year) | | | 37 |
| *Plantago muelleri* above 2000m | N/A | N/A | N/A | N/A | N/A | N/A | ***p< 0.01*** | 0.469 | 0.004 | 40 |
| *Plantago muelleri* below 2000m | N/A | N/A | N/A | N/A | N/A | N/A | 0.412 | 0.031 | 0.001 | 25 |
| Leaf thickness | | | | | | | | | | |
| *Dichosciadium ranunculaceum* var. *ranunculaceum* | 0.241 | 0.069 | -0.259 | 0.486 | 0.071 | 0.057 | 0.695 | 0.081 | 0.001 | 26 |
| *Ozothamnus secundiflorus* | ***0.001*** | 0.333 | -1.500 | ***0.007*** | 0.298 | -0.164 | ***0.001*** | 0.401 | -0.005 | 31 |
| Leaf mass per unit area | | | | | | | | | | |
| *Dichosciadium ranunculaceum* var.*ranunculaceum* | 0.865 | 0.025 | 0.001 | 0.739 | 0.025 | <0.001 | 0.962 | 0.033 | <0.001 | 34 |
| *Grevillea australis* | 0.551 | 0.012 | <0.001 | 0.440 | 0.020 | <0.001 | 0.551 | 0.012 | <0.001 | 32 |
| *Ozothamnus secundiflorus* | 0.122 | 0.078 | -0.001 | 0.112 | 0.079 | <0.001 | 0.122 | 0.083 | -0.001 | 31 |
| *Pentachondra pumila* | 0.240 | 0.041 | 0.001 | 0.209 | 0.045 | <0.001 | 0.240 | 0.043 | 0.001 | 53 |
| *Pimelea alpina* | 0.596 | 0.072 | <0.001 | 0.553 | 0.085 | <0.001 | 0.596 | 0.076 | <0.001 | 36 |
| *Prostanthera cuneata* | 0.515 | 0.019 | <0.001 | 0.644 | 0.013 | <0.001 | 0.515 | 0.020 | <0.001 | 33 |
| Leaf length | | | | | | | | | | |
| *Aciphylla glacialis* | 0.240 | 0.026 | 0.001 | 0.150 | 0.040 | 0.372 | 0.206 | 0.054 | 0.001 | 56 |
| *Microseris lanceolata* | 0.074 | 0.145 | 0.001 | 0.070 | 0.170 | 0.452 | 0.101 | 0.140 | 0.001 | 27 |
| *Oreomyrrhis pulvinifica* | 0.159 | 0.039 | 0.001 | 0.302 | 0.222 | 0.020 | 0.131 | 0.046 | 0.001 | 54 |
| Leaf shape | | | | | | | | | | |
| *Aciphylla simplicifolia* | 0.595 | 0.026 | <0.001 | 0.667 | 0.022 | <0.001 | 0.855 | 0.079 | <0.001 | 27 |
| *Brachyscome spathulata* | 0.556 | 0.125 | <0.001 | 0.849 | 0.124 | <0.001 | 0.457 | 0.170 | <0.001 | 44 |
| *Craspedia costiniana* | 0.335 | 0.020 | 0.001 | 0.370 | 0.016 | <0.001 | 0.318 | 0.030 | -0.001 | 38 |
| *Dichosciadium ranunculaceum* var. *ranunculaceum* | 0.906 | 0.028 | <0.001 | 0.999 | 0.030 | <0.001 | 0.833 | 0.038 | <0.001 | 48 |
| *Drosera arcturi* | **0.010** | 0.149 | -0.002 | **0.012** | 0.130 | -0.001 | **0.004** | 0.292 | -0.003 | 28 |
| *Epacris microphylla* | 0.593 | 0.010 | <0.001 | 0.762 | 0.006 | <0.001 | 0.738 | 0.008 | <0.001 | 25 |
| *Epilobium gunnianum* | 0.706 | 0.004 | <0.001 | 0.690 | 0.005 | <0.001 | 0.506 | 0.023 | <0.001 | 27 |
| *Euphrasia collina* subsp. *diversicolor* | 0.997 | 0.004 | <0.001 | 0.889 | 0.007 | <0.001 | 0.770 | 0.009 | <0.001 | 43 |
| *Gentianella muelleriana* subsp. *alpestris* | 0.957 | 0.068 | <0.001 | 0.972 | 0.055 | <0.001 | 0.877 | 0.060 | <0.001 | 32 |
| *Grevillea australis* | 0.402 | 0.037 | 0.001 | 0.282 | 0.047 | <0.001 | 0.293 | 0.036 | 0.001 | 49 |
| *Ozothamnus secundiflorus* | 0.212 | 0.066 | <0.001 | 0.211 | 0.060 | <0.001 | 0.058 | 0.228 | -0.001 | 27 |
| *Pentachondra pumila* | 0.418 | 0.012 | <0.001 | 0.411 | 0.013 | <0.001 | 0.535 | 0.033 | <0.001 | 40 |
| *Pimelea alpina* | 0.097 | 0.018 | <0.001 | 0.151 | 0.015 | <0.001 | 0.085 | 0.086 | <0.001 | 38 |
| *Plantago muelleri* | 0.394 | 0.031 | -0.001 | 0.343 | 0.035 | <0.001 | 0.434 | 0.047 | <0.001 | 36 |
| *Prostanthera cuneata* | 0.891 | 0.003 | <0.001 | 0.887 | 0.001 | <0.001 | 0.870 | 0.055 | <0.001 | 31 |
| *Ranunculus graniticola* | 0.943 | 0.023 | <0.001 | 0.879 | 0.020 | <0.001 | 0.461 | 0.061 | <0.001 | 25 |
| *Ranunculus niphophilus* | 0.670 | 0.006 | <0.001 | 0.542 | 0.011 | -0.001 | 0.339 | 0.050 | -0.001 | 25 |
| *Senecio pinnatifolius* var. *alpinus* | 0.685 | 0.015 | <0.001 | 0.584 | 0.015 | <0.001 | 0.639 | 0.029 | <0.001 | 45 |
| **Estimated leaf area** | | | | | | | | | | |
| *Dichosciadium ranunculaceum* var. *ranunculaceum* | 0.145 | 0.054 | 0.001 | 0.109 | 0.057 | 0.711 | 0.162 | 0.064 | 0.001 | 48 |
| *Grevillea australis* | 0.500 | 0.024 | <0.001 | 0.335 | 0.038 | 0.023 | 0.465 | 0.043 | <0.001 | 49 |
| *Ozothamnus secundiflorus** | Interaction effect (altitude:year) | | | Interaction effect (altitude:year) | | | Interaction effect (altitude:year) | | | 27 |
| *Ozothamnus secundiflorus* above 1950m | 0.746 | 0.007 | <0.001 | 0.716 | 0.009 | 0.012 | 0.777 | 0.016 | <0.001 | 14 |
| *Ozothamnus secundiflorus* below 1950m | **0.017** | 0.275 | 0.001 | **0.032** | 0.218 | 0.072 | **0.017** | 0.461 | 0.002 | 13 |
| *Pimelea alpina* | 0.570 | 0.003 | <0.001 | 0.596 | 0.013 | <0.001 | 0.638 | 0.007 | <0.001 | 38 |
| *Pentachondra pumila* | 0.861 | 0.009 | <0.001 | 0.960 | 0.008 | <0.001 | 0.636 | 0.077 | <0.001 | 40 |
| *Prostanthera cuneata* | 0.480 | 0.011 | <0.001 | 0.469 | 0.012 | 0.012 | 0.465 | 0.020 | <0.001 | 31 |

#### References

**Buswell JM, Moles AT, Hartley S.** **2011**. Is rapid evolution common in introduced plant species? *Journal of Ecology,* **99**: 214-224.

## Appendix S4. Altitudinal shift over time for *Ozothamnus secundiflorus*

We investigated the change in altitude over time in *O. secundiflorus* due to the interaction effect observed between altitude and year in the analysis of leaf area. We used all observations of this species within 10km of the summit from the Atlas of Living Australia (ALA; <https://www.ala.org.au/>). Contrary to predictions based on warming temperatures, this species has shifted to lower altitudes in more recent years
(p <0.001, R^2^ = 0.311, Fig. S1). Using quantile regression performed in *quantreg* in R (Koenker, 2017) we found that the shift in the distribution of *O. secundiflorus* results from significant decreases in elevation at both the upper and lower edges of the species’ distribution (25^th^ quantile: p < 0.001, slope = -5.681; 75^th^ quantile: p < 0.001, slope =
-1.119).


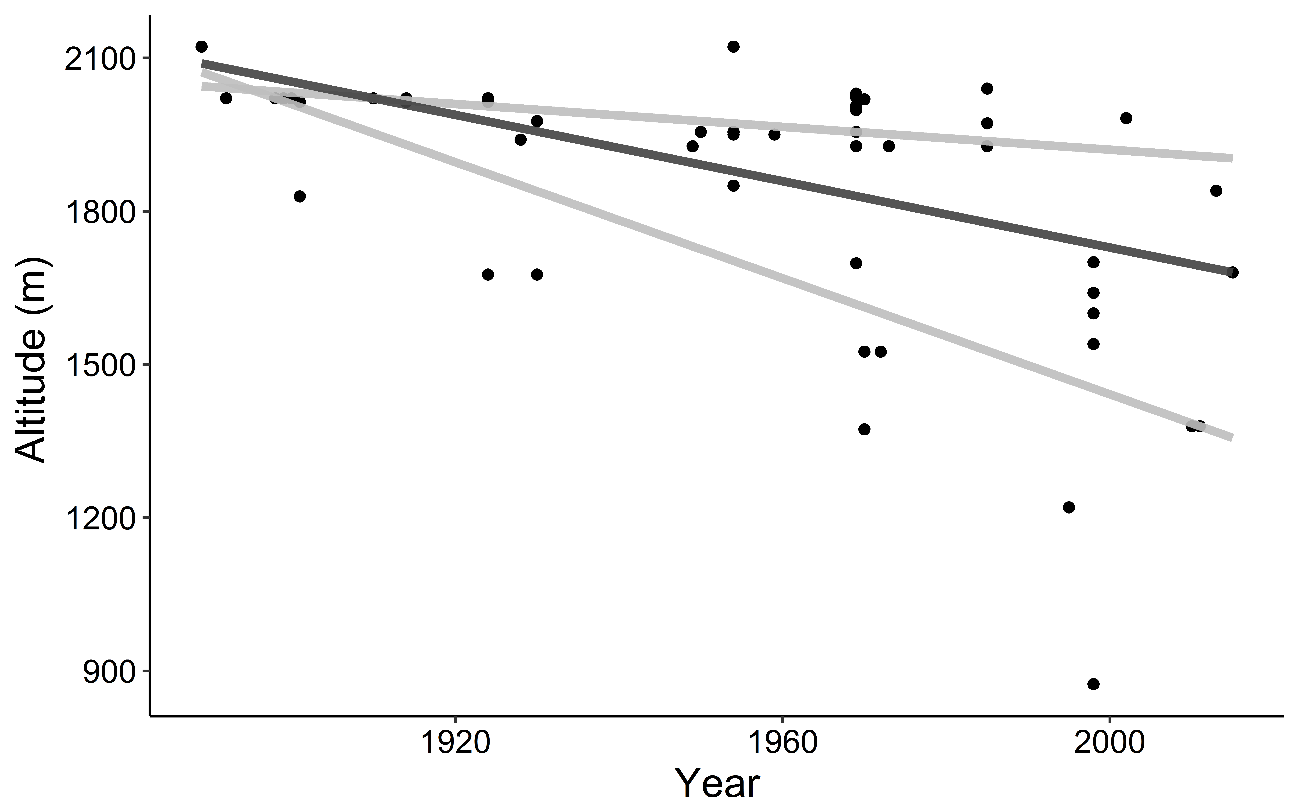

**Figure S1.** Altitudinal distribution of the native alpine plant *Ozothamnus secundiflorus* within a 10km radius from Mount Kosciuszko (2228m) in Kosciuszko National Park. Each data point represents one individual. The ordinal linear regression is marked in dark grey (p <0.001, R^2^ = 0.311). The 25^th^ and 75^th^ quantile regression lines are marked in light grey (25^th^ quantile: p < 0.001, slope = -5.681; 75^th^ quantile: p < 0.001, slope =
-1.119).

#### Reference

**Koenker R.** **2017**. quantreg: Quantile regression. *R package. Version 5.33*. <https://cran.r-project.org/package=quantreg>
